# Quality Risk Management Algorithm for Cold Storage Construction Based on Bayesian Networks

**DOI:** 10.1155/2022/6830090

**Published:** 2022-06-24

**Authors:** Yaping Song, Zhanguo Wei

**Affiliations:** School of Logistics and Transportation, Central South University of Forestry and Technology, Changsha 410004, China

## Abstract

In the cold storage construction project, only by controlling the quality risk of the project can ensure that the cold storage can meet the expected use function and achieve the expected economic benefits after the completion of the cold storage. In order to effectively ensure the key pivot role of cold storage in cold chain logistics, a cold storage construction quality risk management system is constructed to identify and analyze quality risk factors from three dimensions: construction procedures, participating units, and work processes, construct a cold storage construction quality risk evaluation model based on Bayesian network, and through reverse reasoning analysis and sensitivity analysis, key quality risk factors are derived: inadequate quality assurance system, technical delivery is not in place, mismatch of building materials and equipment, inadequate training of skilled workers, completion acceptance is not careful or acceptance standards are unreasonable, and duration does not meet the requirements. Finally, in view of the above quality risks, suggestions and measures are put forward from five aspects: man, material, machine, method, and environment.

## 1. Introduction

In recent years, with the continuous improvement of people's living standards and the rapid development of fresh e-commerce, the demand for fruit and vegetable agricultural products continues to increase. Cold storage is the central link and necessary infrastructure of cold chain logistics, and its construction demand also shows a clear upward trend under the dual promotion of the market and policy. The quality requirements act on the project process at the same time, bringing quality risks into the execution. In addition, cold storage is a complex system facing various unknown risks, so there are more specific and specialized quality risks in the cold storage construction process, which need to be targeted for quality risk management. The external environment of the project is highly variable and unpredictable, coupled with the limitations of human understanding and forecasting ability, which may lead to incomplete cognition and lack of data information in quality risk analysis. The use of Bayesian networks, in this case, is very appropriate because the Bayesian network approach reflects the probabilistic relationship model between the data in the entire database, the lack of a data variable can still build an accurate model. Moreover, the Bayesian network can update the estimated values of all other unknown nodes in the whole network based on the observed node values at any time. For a research object like a cold storage construction project, which is full of uncertainty risks, applying a Bayesian network for quality risk management is in line with the requirements.

After years of development, Bayesian networks have proven their value in many fields, including medical diagnosis, credit evaluation, risk assessment, reliability analysis, prediction, and troubleshooting. A part of experts and scholars used Bayesian networks for various aspects of risk assessment [1–9]. Also, the Delphi method has been utilized along with Bayesian networks to implement risk assessment studies [10, 11]. Some other researchers have combined Bayesian networks with fuzzy set theory in order to perform risk evaluation analysis more easily and quickly [12, 13]. Some experts and scholars used the combination of Bayesian networks and fuzzy hierarchical analysis to achieve their research objectives [14, 15]. Jianxing [16] proposed an intuitive fuzzy Bayesian network-based fault assessment method for process systems in response to imprecise and inadequate historical data. Han [17] constructed a disaster chain hazard assessment model combining the Bayesian network model and ArcGIS program software, then determined hazard chain probabilities and hazard intensities of seismic events using probabilities obtained from Bayesian networks, and produced disaster chain hazard maps using ArcGIS. Li [18] predicted the impacts of land use and climate change on riverine macroinvertebrates based on the linkage of structural equation models with Bayesian networks. Chen and Huang [19] introduced Bayesian networks for flight crew performance assessment to provide data support for interventions in human error management for aviation safety. Mendes [20] used Bayesian networks in context of value-based software engineering to estimate the value of decisions. As for cold storage, Chen [21] prioritized alternative points by analyzing the distribution of cold storage in the northwest region of Zhengming Modern Logistics Company using an improved gray correlation model. Kuźmicki [22] introduced the engineering analysis of cold storage for aquatic products. Chukwu and Adibe [23] performed a quality assessment of cold chain storage facilities for regulatory and quality management compliance in developing countries. Miao and Zhang [24] studied the energy consumption evaluation method and energy-saving operation technology of cold storage.

Not much research has been done on cold storage construction, and in view of this, this paper applies Bayesian networks to develop a study on the quality risk of cold storage construction.

The main contributions of this paper are as follows:

1. Based on the Bayesian network, this paper identifies and corrects the quality risk factor indicators and enriches the theoretical system of quality risk management for cold storage construction projects.
2. This paper gradually changes the quality risk management work from subjective qualitative analysis to visual, quantitative analysis, which is conducive to improving the visualization of quality risk management.
3. It provides a reference for the quality management of cold storage construction projects, which is conducive to improving the construction quality of cold storage.

This paper is organized as follows: Section 2 introduces the quality risk management system for cold storage construction, Section 3 describes the proposed algorithm, Section 4 covers the simulation study, Section 5 presents the experiments and results, and Section 6 concludes the paper.

## 2. Cold Storage Construction Quality Risk Management System

In this paper, we will study the quality risks in the construction process of cold storage engineering projects from three dimensions: construction procedures, participating units, and work processes. Firstly, we identify and analyze the quality risk factors of which each stage and each participating unit needs to bear the corresponding responsibility, use Bayesian networks for structure learning and parameter learning to determine the probability of occurrence of each quality risk, and find out the key quality risk factors through reverse reasoning and sensitivity analysis, and finally propose effective quality risk control measures, in order to be able to provide a reference for the quality management of cold storage construction and to help achieve the goal of safe production, so as to give full play to the role of the cold storage itself and ensure the normal operation of the storage and preservation function.

### 2.1. Construction Procedures

Considering the complexity of the research object of this paper and taking into account the actual situation, this paper will carry out the research process in four stages: decision-making stage, design work stage, engineering construction stage, and completion acceptance and delivery stage, and combine the four stages to analyze the quality risk factors from the whole process. By reading the relevant literature, it can be concluded that the main quality risks existing in each stage are shown in Table 1.

### 2.2. Participating Units

Quality risk management of engineering construction projects reflects the characteristics of diversified subjects. Construction units, design units, building units, supervision units, etc., are responsible for the quality risk management of engineering construction projects. Therefore, this paper delineates the participating units in cold storage construction as construction units, design units, building units, and supervision units and studies the quality risk factors that exist in terms of the dimensions of the participating units. A review of the literature shows that the main quality risk factors that may exist in the participating units in cold storage construction are shown in Table 2.

### 2.3. Work Processes

From the perspective of the work content involved in the cold storage construction project, there are a series of interrelated and mutually influencing workflows that require quality risk management from the decision-making stage to the completion acceptance and delivery stage. In order to ensure the smooth progress of each work process, it is necessary to analyze the quality risk factors that may exist in the process. According to relevant references, the quality risk factors existing in the cold storage construction work processes are shown in Table 3.

## 3. Proposed Algorithm

The seminal work on Bayesian networks is the book written by British mathematician Thomas Bayes: *An Essay toward Solving a Problem in the Doctrine of Chances.* In 1958, the British statistical journal *Biometrika* republished the Bayesian paper in full. In the 1950s, the combination of empirical Bayesian methods with classical methods in the estimation of econometric models, represented by H. Robbins, attracted widespread attention. In 1985, Judea Pearl first proposed the Bayesian network model and applied it to artificial intelligence aspects for probabilistic inference.

### 3.1. Bayesian Theory

The process of Bayes' theorem can be summarized as follows: “past experience” plus “new evidence” yields “modified judgment.” It provides an objective method of combining newly observed evidence with existing experience to make inferences.

Suppose there are random events *A* and *B*, their conditional probability relationship can be expressed by the following mathematical formula:

(1) $$\begin{array}{c} P\left(B|A\right)=P\left(B\right)\frac{P\left(A|B\right)}{P\left(A\right)}. \end{array}$$

The event *B* is the target event to be examined and *P*(*B*) is the initial probability of the event *B*, called the prior probability. *A* is a newly emerged event that will affect event *B*, *P*(*A*) denotes the probability of event *A* occurring. *P*(*A|B*) denotes the probability of *A* when *B* occurs, which is a conditional probability, and *P*(*B|A*) denotes the probability of *B* when *A* occurs, which is both a conditional and a posteriori probability.

According to the Bayesian formula, the prior probability is generally the probability data obtained from previous data analysis or statistics. The posterior probability is the probability of occurrence under certain conditions and is the probability that is re-corrected after the information is obtained. In other words, the posterior probability can be corrected and obtained on the basis of the prior probability.

Suppose *B*_1_, *B*_2_,…, *B*_*i*_ is a division of the sample space Ω, then for any event *A*(*P*(*A*) > 0), there is the following:

(2) $$\begin{array}{c} P\left(B_{i}|A\right)=\frac{P\left(B_{i}\right)P\left(A|B_{i}\right)}{\sum_{j=1}^{n}P\left(B_{j}\right)P\left(A|B_{j}\right)}. \end{array}$$

The above formula is the Bayes formula, *B*_*i*_ is often regarded as the cause of the test result *A*, *P*(*B*_*i*_)(*i*=1,2,…) indicates the probability of various causes, so it is called the prior probability; *P*(*B*_*i*_*|A*) reflects the new understanding of the probabilities of various causes after the test has produced results, so it is called the posterior probability.

### 3.2. Definition

**Figure 1:**
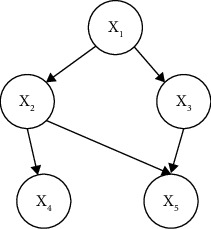
Directed acyclic diagram.

A Bayesian network is a causal network graph, which mainly consists of two parts: a directed acyclic graph and a conditional probability table. A directed acyclic graph is a non-closed relational graph with directionality, consisting of several nodes and directed arcs between nodes. The details are shown in Figure 1. The nodes represent random variables in the knowledge domain, and the directed arcs represent the interdependencies between the nodes, which are directed from the parent nodes to the child nodes. The conditional probability table is a quantitative description of the dependencies between the node variables. Its expression is as follows:

(3) $$\begin{array}{c} F_{BN}=\langle G,p\left(x\right)\rangle . \end{array}$$

*G*=(*I*, *E*) denotes a directed acyclic graph, where *I* represents the set of all points in the graph and *E* represents the set of directed connected line segments. And let *X*=(*X*_*i*_)_*i*∈*I*_ be the random variable represented by a node *i* in its directed acyclic graph, if the joint probability distribution of the variable *X* can be denoted as *p*(*x*)={*p*(*X*_*i*_*|X*_*pa*(*i*)_}, namely the conditional probability table is *p*(*x*), where pa(*i*) denotes the cause of node *i*, or pa(*i*) is the parents of *i*.

Furthermore, for any random variable, its joint probability can be obtained by multiplying the respective local conditional probability distributions:

(4) $$\begin{array}{c} p\left(x_{1},\cdots ,x_{i}\right)=p\left(x_{i}|x_{1},\cdots ,x_{i-1}\right)\cdot \cdots \cdot p\left(x_{2}|x_{1}\right)p\left(x_{1}\right). \end{array}$$

### 3.3. Characteristics

The Bayesian network can use the knowledge obtained under uncertain and complex conditions to infer logical results and has the ability of system modeling, reasoning, and diagnosis. It is the most effective way to deal with event polymorphism and uncertainty in data mining technology. As an ideal analysis tool, the Bayesian network based on probabilistic reasoning has great advantages in representing and solving decision-making problems with uncertain factors and can solve the faults caused by the uncertainty and correlation of complex equipment, so it can be used in multiple widely used in the field.

The Bayesian network has the following advantages: the estimated values of all other unknown nodes in the entire network can be updated at any time according to the observed node values; the causal relationship between nodes can be displayed intuitively, and modeling can be based on expert knowledge in the case of insufficient data; two-way reasoning can be performed, either from the cause to the result or from the result to the cause. Such reverse reasoning ability cannot be achieved by other classical probabilistic reasoning methods; reasoning can be done in the case of incomplete data because the Bayesian network method reflects the probability relationship model between the data in the entire database; it can combine multiple types of data, such as subjective empirical data and objective data; the results of quality risk analysis are given in probabilistic form, which is more intuitive and reliable.

### 3.4. Bayesian Network Structure Learning

Bayesian network structure learning refers to the determination of a structural model, namely a directed acyclic graph, by experts in the relevant field based on the relationships between things. The selection of a suitable and appropriate structure learning method helps to construct the optimal Bayesian network structure model. There are three general Bayesian network structure learning methods: First, based on expert knowledge construction, using the empirical knowledge of experts in related fields to analyze the connection relationship between nodes and determine a Bayesian network structure that meets reality. Second, based on data learning, the Bayesian network topology is obtained by training on previous sample data. Third, the sequence of nodes is determined based on expert knowledge and experience, and the structure of the Bayesian network is determined with a suitable algorithm. Due to the specificity of the research object and the restriction of the sample size, this paper chooses to construct the Bayesian network topology based on expert knowledge and experience combined with the questionnaire survey.

### 3.5. Bayesian Network Parameter Learning

Bayesian network parameter learning refers to determining the conditional probability distribution at each node of a Bayesian network model for given Bayesian network topology, using the expert's prior knowledge combined with the actual situation. For Bayesian network parameter learning, there are two common methods: the maximum likelihood estimation method and the Bayesian statistics method. Both methods need to meet the sample data independently and identically distributed, and the biggest difference is that the estimated parameters are different. Among them, the maximum likelihood estimation method regards the parameter to be estimated as a fixed form of an unknown variable, and the solution process is not constrained by previous knowledge and experience. In contrast, the Bayesian statistical method regards the parameter as a random variable with some known prior distribution and can refer to previous knowledge and experience. Given that the research object of this paper is a cold storage construction project, we need to fully cross-reference similar engineering projects and draw on the relevant experience of engineers, so we choose Bayesian statistics for parameter learning of Bayesian networks in this paper.

## 4. Simulation Study

### 4.1. Risk Identification

In this paper, a questionnaire survey was issued to relevant professionals in the industry to achieve the identification of quality risk factors in cold storage construction projects, the partial list of which is shown in Table 4. Practitioners include project managers, designers, construction technicians, supervision engineers, etc., who have rich experience in cold storage construction. The main content of the questionnaire is divided into the probability of occurrence of quality risks in cold storage construction projects and the degree of impact of the risks, which are divided into five levels.

### 4.2. Risk Analysis and Assessment

**Figure 2:**
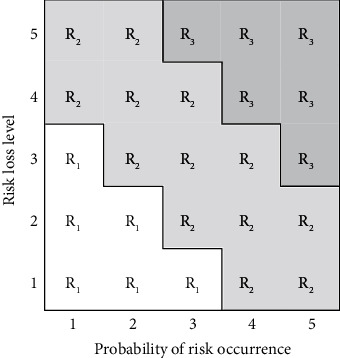
Risk level matrix.

Risk analysis and assessment is the process of measuring the risks of the identified project processes and project products. The main components include determining the probability of risk occurrence and the severity of impact on project objectives, evaluating the potential impact of all risks, and thus obtaining the values of risk decision variables for the project, which are used as an important basis for project decision making. Each risk can be measured in terms of its probability of occurrence and potential loss value and can also be analyzed with the help of a risk level matrix, as shown in Figure 2, where different grids represent different amounts of risk with *R*_1_ representing low risk, *R*_2_ representing medium risk, and *R*_3_ representing high risk. The horizontal axis indicates the probability of risk occurrence, and the vertical axis indicates the degree of risk loss, both with five levels. The expected value of risk is equal to the probability of risk occurrence multiplied by the risk loss level. The risk classification table for cold storage construction quality is shown in Table 5.

Statistically, 72 questionnaires were returned, of which 58 were valid. The data in the questionnaires were processed and the quality risk factors with *R*_3_ ratio of 15% and above were selected as key indicators. The statistical results are shown in Table 6.

## 5. Experiments and Results

### 5.1. Bayesian Network Model

In this paper, Netica is used for simulation experiments. Netica is currently the most widely used Bayesian network analysis software in the world, featuring simplicity, reliability, and efficiency. Netica Application is a comprehensive tool for working with Bayesian belief nets and decision nets (influence diagrams). It can build, learn, modify, transform and store nets, as well as answer queries or find optimal solutions using its powerful inference engine. As a decision-making tool, it is widely used in business, engineering, medical and ecological analysis.

By consulting relevant materials and asking experts and scholars for their opinions, the following quality risk causality chains are drawn:

① Improper coordination between the construction unit and the design, building, and supervision parties (*E*1) ⟶ Insufficient financing (*A*3)⟶ Unqualified quality of building materials and equipment (C15) ⟶ Cold storage technology is not applicable (*C*12)
② Improper coordination between the construction unit and the design, building, and supervision parties (*E*1) ⟶ Frequent design changes (*B*3)⟶ Construction technology is difficult (*C*11) ⟶ Duration does not meet the requirements (*C*5)
③ Inadequate quality assurance system (*E*2) ⟶ Technical delivery is not in place (*C*8) ⟶ Inadequate training of skilled workers (*C*13) ⟶ Completion acceptance is not careful or acceptance standards are unreasonable (*D*1)
④ Inadequate quality assurance system (*E*2) ⟶ Mismatch of building materials and equipment (*C*16) ⟶ Cold storage technology is not applicable (*C*12)
⑤ Inadequate quality assurance system (*E*2) ⟶ Supervision is not effective (*E*5) ⟶ Completion acceptance is not careful or acceptance standards are unreasonable (*D*1)
⑥ Construction drawings are not designed to meet construction and billing requirements and do not take into account construction possibilities (*B*1) ⟶ The preparation of the feasibility study report is unreasonable (*A*2) ⟶ Backwardness of the construction process (*C*10)
⑦ Construction drawings are not designed to meet construction and billing requirements and do not take into account construction possibilities (*B*1) ⟶ Impact of force majeure (*C*14) ⟶ Construction technology is difficult (*C*11) ⟶ Duration does not meet the requirements (*C*5)
⑧ Inadequate construction preparation (*C*1) ⟶ Unfavorable project environment (*E*4) ⟶ Construction technology is difficult (*C*11) ⟶ Duration does not meet the requirements (*C*5)

**Figure 3:**
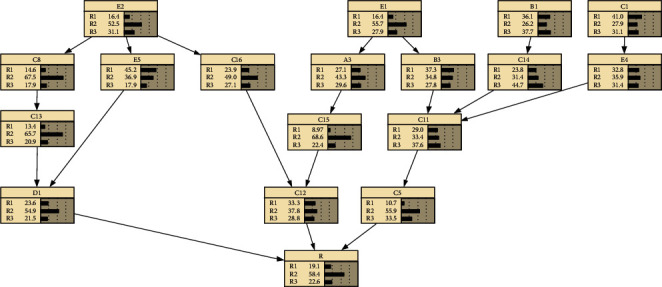
Bayesian network of cold storage construction quality risks.

According to the above causal relationship chain, a Bayesian network topology is constructed in Netica software, the questionnaire data is made into a table, and the table data is imported for parameter learning, and finally a complete Bayesian network model is presented, as shown in Figure 3.

Finally, through the established Bayesian network of quality risk of cold storage construction, it can be concluded that the overall quality risk level of cold storage construction is shown in Table 7 below.

### 5.2. Bayesian Network Diagnostic Inference Analysis

#### 5.2.1. Reverse Reasoning

**Figure 4:**
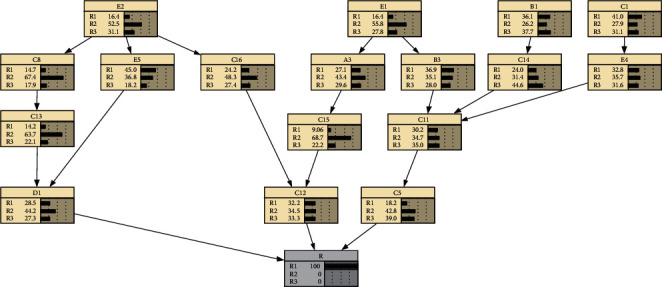
Bayesian network reverse reasoning of cold storage construction quality risks (*R*_1_).

Reverse reasoning refers to assuming that the probability of occurrence of a child node is 100%, and reversely infers the probability of occurrence of the corresponding parent node. Assuming that the occurrence level of the total quality risk of cold storage construction is *R*_1_; that is, the probability of occurrence of *R*_1_ is 100%, the following Bayesian network can be obtained as shown in Figure 4.

The comparative analysis shows that, compared with the original Bayesian network, when the overall quality risk level of the cold storage construction is assumed to be *R*_1_, the risk occurrence probability of *D*1 and *C*5 has changed significantly, the probability of *R*_2_ is reduced, and the probability of *R*_1_ and *R*_3_ increases. This shows that when the overall quality risk level of cold storage construction is *R*_1_, the most affected factors are duration does not meet the requirements and completion acceptance is not careful or acceptance standards are unreasonable.

**Figure 5:**
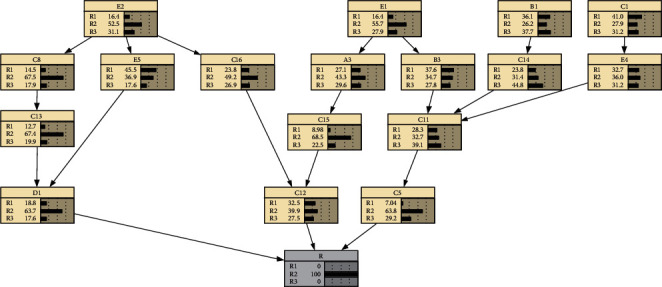
Bayesian network reverse reasoning of cold storage construction quality risks (*R*_2_).

Assuming that the occurrence level of the total quality risk of cold storage construction is *R*_2_; that is, the probability of occurrence of *R*_2_ is 100%, the following Bayesian network can be obtained as shown in Figure 5.

The comparative analysis shows that compared with the original Bayesian network, the probability of risk occurrence of *D*1 and *C*5 appears to have more obvious changes when assuming that the level of the overall quality risk of cold storage construction is *R*_2_, showing an increase in the probability of *R*_2_ and a decrease in the probability of occurrence of *R*_1_ and *R*_3_.

**Figure 6:**
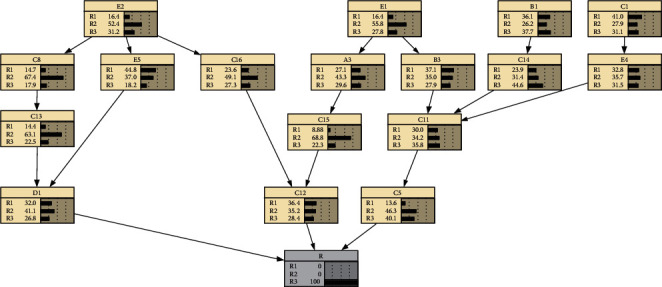
Bayesian network reverse reasoning of cold storage construction quality risks (*R*_3_).

Assuming that the occurrence level of the total quality risk of cold storage construction is R_3_; that is, the probability of occurrence of *R*_3_ is 100%, the following Bayesian network can be obtained as shown in Figure 6.

The comparative analysis shows that compared with the original Bayesian network, the probability of risk occurrence of *D*1 and *C*5 appears to have more obvious changes when assuming that the level of the overall quality risk of cold storage construction is *R*_3_, showing a decrease in the probability of *R*_2_ and an increase in the probability of occurrence of *R*_1_ and *R*_3_.

In summary, when different levels of quality risks occur in cold storage construction, the corresponding risk levels of other nodes will increase, with the probability of *C*5 (Duration does not meet the requirements) and *D*1 (Completion acceptance is not careful or acceptance standards are unreasonable) changing more significantly, indicating that these two factors have a greater impact on the overall quality risk of cold storage construction.

#### 5.2.2. Sensitivity Analysis

**Figure 7:**
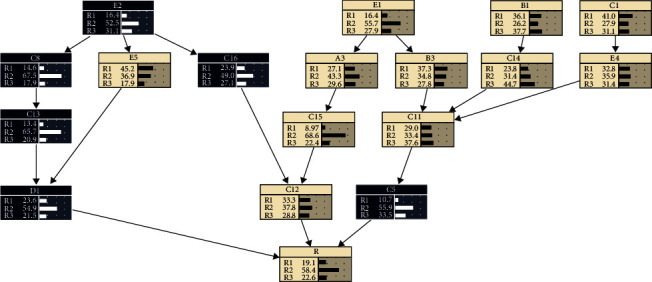
Bayesian network sensitivity analysis of cold storage construction quality risks.

Sensitivity analysis refers to finding out the key nodes that are greatly affected by other nodes by observing the degree of influence of each node by other nodes and using the correlation effect between nodes. Select the “Sensitivity to Findings” operation in the Netica software, and then check the influence of other discovery nodes in the network on selecting different target nodes. The sensitive factors that are most affected are listed below. They are *E*2 (Inadequate quality assurance system), *C*8 (Technical delivery is not in place), *C*16 (Mismatch of building materials and equipment), *C*13 (Inadequate training of skilled workers), *D*1 (Completion acceptance is not careful or acceptance standards are unreasonable), and *C*5 (Duration does not meet the requirements). The specific nodes are shown in Figure 7.

The sensitivity analysis of *E*2 is shown in Table 8. The main influencing factors of *E*2 (Inadequate quality assurance system) are *E*5 (Supervision is not effective), *C*16 (Mismatch of building materials and equipment), and *C*8 (Technical delivery is not in place).

The sensitivity analysis of C8 is shown in Table 9. It can be seen that *C*8 (Technical delivery is not in place) is mainly affected by *C*13 (Inadequate training of skilled workers) and E2 (Inadequate quality assurance system).

The sensitivity analysis of *C*16 is shown in Table 10. *C*16 (Mismatch of building materials and equipment) is mainly affected by *E*2 (Inadequate quality assurance system) and *C*12 (Cold storage technology is not applicable).

The sensitivity analysis of *C*13 is shown in Table 11. It can be seen that *C*13 (Inadequate training of skilled workers) is mainly affected by *C*8 (Technical delivery is not in place) and *D*1 (Completion acceptance is not careful or acceptance standards are unreasonable).

The sensitivity analysis of *D*1 is shown in Table 12. It can be seen that *D*1 (Completion acceptance is not careful or acceptance standards are unreasonable) is mainly affected by *C*13 (Inadequate training of skilled workers).

The sensitivity analysis of *C*5 is shown in Table 13. It can be seen that *C*5 (Duration does not meet the requirements) is mainly affected by *C*11 (Construction technology is difficult).

The sensitivity analysis of *R* is shown in Table 14. The main influencing factors of *R* (Cold storage construction quality risk) are *D*1 (Completion acceptance is not careful or acceptance standards are unreasonable) and *C*5 (Duration does not meet the requirements).

A quality assurance system is a key part of quality management and risk management, which is sensitive to the construction of cold storage; technical delivery is the preparation work before construction so that the construction personnel understands all aspects of the project in detail so as to facilitate the scientific organization of construction and avoid accidents, so this link is also a sensitive factor; construction materials and equipment are indispensable in the construction process, and its quality is crucial to the successful completion of the project; skilled workers are the main implementers of the construction, and the quality of the operators will directly affect the construction of the cold storage; completion acceptance is the assessment and inspection of the project, and is the last line of defense to regulate the quality of the building. Duration is one of the important accounting indicators of construction enterprises, and the length of duration directly affects the economic benefits of construction enterprises. Reasonable arrangement of duration and organization of flow work can improve labor productivity and reduce engineering costs. In summary, the nodes identified by Bayesian network sensitivity analysis are all sensitive factors in the cold storage construction process, which need to be monitored by key prevention.

### 5.3. Quality Risk Management Measures

After the Bayesian network reverse reasoning analysis and sensitivity analysis, this article provides relevant quality risk management measures based on the above analysis results and add from after explains five aspects of man, material, machine, method, and environment.

#### 5.3.1. Measures on the Man Aspect

Man is the main force of implementation. To ensure the smooth progress of construction projects, standardized management measures need to be taken for technical workers. First of all, focus on construction related training to ensure that workers are proficient in relevant basic operation steps, and organize technical workers to conduct technical intercourse, so that they can learn more about the entire project in order to effectively organize scientific construction and improve work efficiency. At the same time, regularly organize technical assessment, screen qualified workers, and give workers a sense of urgency, pressure, and responsibility.

As a party on behalf of the construction unit, the supervision unit shall strengthen the professionalism of the supervision engineer and organize relevant training on a regular basis. It is necessary to ensure that the supervision engineer must be serious, professional, and responsible in the process of engineering construction to ensure the smooth completion of the cold storage.

#### 5.3.2. Measures on the Material Aspect

In order to grasp the quality off and quantity off, materials and equipment need to be inspected and accepted according to the incoming material plan, acceptance specifications, etc., when they come in, and records should be made. When the materials are put into the warehouse, a ledger should be established, reasonably placed, and regularly inventoried to ensure that the accounts match. When receiving and issuing materials, it is necessary to receive and issue materials within the limit, to indicate the reason for using materials beyond the limit, and to establish a ledger to record the receiving and issuing situation. The person responsible for on-site material management should supervise the use of on-site materials to ensure that the materials are used reasonably according to the material plan. When the surplus material is recycled, the material will be returned in time and the subsequent use process will be reasonably arranged.

In view of the particularity of the cold storage, in order to realize the normal operation of the thermal insulation function of the cold storage, it is necessary to purchase qualified thermal insulation materials, to achieve good thermal insulation and moisture resistance, and to adapt to local conditions and make full use of them.

#### 5.3.3. Measures on the Machine Aspect

The selection of construction machinery should follow the principles of relevance to needs, practical possibility, and economic reasonableness. When choosing construction machinery, first choose the leading project machinery, according to the characteristics of the project, to choose the most suitable type. At the same time, in order to give full play to the efficiency of leading machinery, the corresponding auxiliary facilities should be selected accordingly to coordinate their production capacity.

Construction machinery and equipment can only be admitted after acceptance, and the maintenance responsibility system is implemented during use, with dedicated maintenance and repair to maintain the good technical condition of the equipment, improve the reliability and safety of equipment operation, reduce losses, and improve economic efficiency. The operators of the equipment must be trained uniformly and can only take up their posts after passing the test. Regularly organize skill examinations to make technical personnel proficient in equipment operation skills and ensure that the equipment is used reasonably. Compile machinery and equipment use plans scientifically, and consider when using a certain construction process, the use of what and how to use machinery and equipment is the most reasonable and efficient.

#### 5.3.4. Measures on the Method Aspect

Whether the implementation plan is reasonable, the process operation is correct, and the technology is advanced, all of these can have a big impact on the project. The selection of construction machinery and construction methods should be unified and coordinated. When choosing the construction method, it should focus on the construction method of the component works that affect the construction of the whole project.

Cold storage should have a reasonable structure and good heat insulation to ensure the quality of food storage. Cold storage mainly consists of an enclosure structure and load-bearing structure, of which the enclosure structure should have good heat insulation and moisture-proof effect to resist the storm outside the storage, and the load-bearing structure should be able to support the weight of goods and loading and unloading equipment. As a productive power-using unit, energy-saving measures should be taken to reduce energy consumption in cold storage. The heat transfer of the enclosure structure accounts for 20%–35% of the total heat load of the cold storage, so the construction process needs to ensure the good performance of the cold storage enclosure structure.

Use project management software appropriately for time scheduling, resource management, cost management, project monitoring, and information sharing, etc. Information technology can propose a variety of options for decision makers in response to project characteristics, making project management work fast and thus saving a lot of human resources.

Improve the quality assurance system. Decompose the quality objectives into specific tasks and implement them in each department with clear responsibilities. Establish a quality management system and document it for implementation, maintenance, and continuous improvement. According to the characteristics of different projects, on the basis of the original quality management system, more targeted quality plans and quality procedures are formulated. During the operation of the system, regular supervision and assessment are carried out, and the effectiveness and suitability of the system are promoted through internal and external review.

#### 5.3.5. Measures on the Environment Aspect

Reasonably arrange the progress of the project to avoid the influence of bad weather. Investigate the geological and hydrological conditions required by the cold storage project in advance. Improve the working environment of project operators, provide good office conditions, office facilities, communication conditions, and security to improve labor productivity. Implement the environmental protection target responsibility system and assign environmental protection responsibility to departments or personnel. Establish and operate an environmental management system efficiently, mobilize the enthusiasm of relevant on-site organizations, and implement environmental inspection and monitoring. Formulate emergency measures to maintain a good working environment, sanitary conditions, and public order on-site, and make continuous improvements.

According to the principle of purpose, set up affairs according to the goals, set up institutions and staff according to the situation, set up positions according to the staff, set up systems, and delegate powers according to responsibilities; according to the principle of lean and efficient, try to simplify the organization as much as possible, so that people can make the best use of their talents. Through the establishment of a highly efficient organization that operates freely, a responsibility system and an information communication system are formed so as to achieve a reasonable division of labor and collaboration. The production activities of construction projects are staged, open-air and fluid, and management work and organizational structures need to be adjusted accordingly to adapt to changes in construction tasks.

## 6. Conclusion

This paper starts with the research on the quality risk management of cold storage construction projects from three aspects: construction procedures, participating units and work processes. First, analyze and identify the quality risk factors existing in the four stages of the decision-making stage, design work stage, engineering construction stage, completion acceptance and delivery stage, analyze the whole process by integrating the four stages, and correspond to the relevant responsible units, then carry out relevant research according to the risk management process, and build a Bayesian network model of cold storage construction quality risk. Through reverse reasoning analysis and sensitivity analysis, the key quality risk factors are obtained: inadequate quality assurance system, technical delivery is not in place, mismatch of building materials and equipment, inadequate training of skilled workers, completion acceptance is not careful, or acceptance standards are unreasonable, and duration does not meet the requirements. Finally, in view of the above-mentioned quality risks, suggestions and measures are put forward from five aspects: man, material, machine, method, and environment. The quality risk management system of cold storage construction in this paper makes full use of the advantages of the Bayesian network in quality risk assessment and realizes the application of the Bayesian network for the complex engineering project of cold storage construction so as to conduct related research more effectively.

## Figures and Tables

**Table 1:** Quality risk factors of cold storage construction procedures.

Construction procedures	Quality risk factors
Decision-making stage	Project proposals and feasibility studies are not reasonable
Design work stage	Incomplete design content, defective design, errors and omissions, inappropriate specifications, failure to consider geological conditions, failure to consider construction possibilities, etc.
Engineering construction stage	Backward construction techniques, unreasonable construction techniques, and solutions, improper construction safety measures, failure to apply new technologies and solutions, failure to consider site conditions, etc.
Completion acceptance and delivery stage	Operation and maintenance risks
Full stage	Improper coordination of the parties involved

**Table 2:** Quality risk factors of the units involved in cold storage construction.

Participating units	Quality risk factors
Construction units	Poor investor decisions
Design units	The professional quality of the design party is not high
Building units	Construction level is not in accordance with the specifications
Supervision units	Inadequate supervision by the supervisor

**Table 3:** Quality risk factors in cold storage construction work processes.

Work processes	Quality risk factors
Preparation of technical data and documents	Inadequate survey information
	Unqualified construction organization design document preparation
Design delivery and drawing review	Inadequate design delivery and drawing review
Procurement and subcontracting	Material procurement does not meet specifications
	Inadequate control of subcontracting services
Technical presentation	Inadequate technical presentation
Engineering surveying	Deviations in measurement results
Completion and acceptance	Unreasonable acceptance criteria

**Table 4:** Partial list of quality risk factors of cold storage construction.

Construction procedures	Participating units	Work processes
*A*-decision-making stage	Construction units	*A*1-unreasonable preparation of project proposal
		*A*3-insufficient financing
	…	…
*B*-design work stage	Design units	*B*1-construction drawings are not designed to meet construction and billing requirements and do not take into account construction possibilities
		*B*3-frequent design changes
	…	…
*C*-engineering construction stage	Building units	*C*1-inadequate construction preparation
		*C*2-construction does not meet completion standards
	…	…
*D*-completion acceptance and delivery stage	Construction units, design units, building units, supervision units	D1-completion acceptance is not careful or acceptance standards are unreasonable
		Acceptance standards are unreasonable
	Building units	*D*2-the technical information is not complete
	…	…
*E*-full stage	Supervision units	*E*5-supervision is not effective
	…	…

**Table 5:** Classification of quality risk levels of cold storage construction.

Risk level	Nature of risk	Measures
*R*_1_	Low risk	Monitoring is required and no action is required
*R*_2_	Medium risk	Efforts are made to reduce risks and monitoring is required
*R*_3_	High risk	Monitoring is required and work will not start until the risk has been reduced

**Table 6:** Key quality risk factors of cold storage construction.

Construction procedures	Participating units	Work processes	Statistical result of risk level (%)		
			*R*_1_	*R*_2_	*R*_3_
*A*-decision-making stage	Construction units	*A*2-the preparation of the feasibility study report is unreasonable	10	75	15
		*A*3-insufficient financing	26	45	29
*B*-design work stage	design units	*B*1-construction drawings are not designed to meet construction and billing requirements and do not take into account construction possibilities	36	26	38
		*B*3-frequent design changes	37	35	28
*C*-engineering construction stage	Building units	*C*1-inadequate construction preparation	42	27	31
		*C*5-duration does not meet the requirements	6	61	33
		*C*8-technical delivery is not in place	11	73	16
		*C*10-backwardness of the construction process	32	42	26
		*C* 11-construction technology is difficult	24	32	44
		*C*12-cold storage technology is not applicable	31	42	27
		*C*13-inadequate training of skilled workers	10	71	19
		*C*14-impact of force majeure	23	31	46
		*C*15-unqualified quality of building materials and equipment	5	74	21
		*C*16-mismatch of building materials and equipment	23	52	25
*D*-completion acceptance and delivery stage	Construction units, design units, building units, supervision units	*D*1-completion acceptance is not careful or acceptance standards are unreasonable	21	62	17
*E*-full stage	Construction units, design units, building units, supervision units	*E*1-improper coordination between the construction unit and the design, building and supervision parties	16	57	27
		*E*2-inadequate quality assurance system	16	54	30
		*E*4-unfavorable project environment	33	36	31
	Supervision units	*E*5-supervision is not effective	47	38	15

**Table 7:** Quality risk levels of cold storage construction.

Risk level	Probability (%)
*R*_1_	19.1
*R*_2_	58.4
*R*_3_	22.6

**Table 8:** Sensitivity of *E*2 to a finding at another node.

Node	Mutual info	Percent	Variance of beliefs
*E*2	1.44008	100	0.3808160
*E*5	0.11391	7.91	0.0248665
*C*16	0.06810	4.73	0.0064580
*C*8	0.03475	2.41	0.0038769

**Table 9:** Sensitivity of *C*8 to a finding at another node.

Node	Mutual info	Percent	Variance of beliefs
*C*8	1.23280	100	0.2986122
*C*13	0.03734	3.03	0.0068553
*E*2	0.03475	2.82	0.0052657

**Table 10:** Sensitivity of *C*16 to a finding at another node.

Node	Mutual info	Percent	Variance of beliefs
*C*16	1.50800	100	0.4097652
*E*2	0.06810	4.52	0.0050632
*C*12	0.02476	1.64	0.0035677

**Table 11:** Sensitivity of *C*13 to a finding at another node.

Node	Mutual info	Percent	Variance of beliefs
*C*13	1.25863	100	0.3085228
*C*8	0.03734	2.97	0.0021347
*D*1	0.02978	2.37	0.0067237

**Table 12:** Sensitivity of *D*1 to a finding at another node.

Node	Mutual info	Percent	Variance of beliefs
*D*1	1.44373	100	0.3820662
*R*	0.03218	2.23	0.0069764
*C*13	0.02978	2.06	0.0062238

**Table 13:** Sensitivity of *C*5 to a finding at another node.

Node	Mutual info	Percent	Variance of beliefs
*C*5	1.34205	100	0.3420480
*R*	0.03069	2.29	0.0059037
*C*11	0.02718	2.03	0.0068624

**Table 14:** Sensitivity of *R* to a finding at another node.

Node	Mutual info	Percent	Variance of beliefs
*R*	1.39406	100	0.3614260
*D*1	0.03218	2.31	0.0072761
*C*5	0.03069	2.2	0.0064688

## Data Availability

The data used to support the findings of this study are available from the corresponding author upon request.
